# A multi-view RGB image dataset of four potato varieties with visually assessed scab and dry-rot severity under dual-background imaging conditions

**DOI:** 10.1016/j.dib.2026.113375

**Published:** 2026-10-23

**Authors:** Samiul Islam Saif, Mst. Fahmida Tasnim Saba, M. Shamim Kaiser

**Affiliations:** a Department of Computer Science, American International University-Bangladesh, Kuratoli, Khilkhet, Dhaka 1229, Bangladesh; b Faculty of Agriculture, Hajee Mohammad Danesh Science and Technology University, Dinajpur, Bangladesh; c Institute of Information Technology, Jahangirnagar University, Savar, Dhaka 1342, Bangladesh

**Keywords:** Agricultural computer vision, Tuber phenotyping, Postharvest quality, Smartphone acquisition, Cold storage, Machine learning

## Abstract

This data article describes a multi-view collection of 9106 RGB JPG images of potato tubers from four varieties: Rumana, BARI Alu-25 (Asterix), Lal Pakri, and BARI Alu-7 (Diamant). The source photographs were acquired at New Kafela Cold Storage, Shibganj, Bogura, Bangladesh, during two approximately one-week collection campaigns beginning on 8 November 2025 and 22 July 2026. Two smartphones, iQOO Neo 11 and realme Narzo 50, were used under natural daylight at an approximate camera-to-tuber distance of 15–20 cm. Source photographs were captured at approximately 4096 × 3072 pixels in JPG format. Each physical tuber was photographed from five orientations (top, bottom, middle, left, and right) on two backgrounds (white paper and concrete floor), giving up to ten photographs per tuber when all views were retained. For public repository release, the retained images were standardized to 512 × 512 pixels while preserving aspect ratio and padding the remaining area. All labels were assigned manually by the first author through visual phenotypic assessment. Rumana is represented by Healthy, Scab-Mild, Scab-Moderate, and Scab-Severe classes. Asterix, Lal Pakri, and Diamant are represented by Healthy and DryRot-Mild, DryRot-Moderate, and DryRot-Severe classes, with Fully-Rotten classes additionally included for Asterix and Diamant. The final release contains 18 combined variety-condition classes and preserves the observed class frequencies. The data can be reused for agricultural computer vision, tuber phenotyping, class-imbalance studies, viewpoint and background analysis, and development of image-based potato condition-recognition methods.

Specifications Table

**Table:** 

Subject	Computer Science
Specific subject area	Agricultural computer vision and image-based phenotyping of potato tubers
Type of data	Processed RGB images (JPG; 512 × 512 pixels); curated class-count table (CSV); resize-processing manifest (CSV); figures (PNG).
Data collection	Potato tubers were photographed with iQOO Neo 11 and realme Narzo 50 smartphones under natural daylight at an approximate 15–20 cm distance. Source JPG images were captured at approximately 4096 × 3072 pixels. Four varieties were photographed from five orientations on white-paper and concrete-floor backgrounds. Labels were assigned manually by visual phenotypic assessment. For public release, retained images were EXIF-corrected, converted to RGB, resized with aspect ratio preserved, padded, and saved at 512 × 512 pixels.
Data source location	New Kafela Cold Storage, Shibganj, Bogura, Bangladesh. Acquisition campaigns began on 8 November 2025 and 22 July 2026 and each continued for approximately one week.
Data accessibility	Repository name: Mendeley Data Data identification number / doi:10.17632/rgn2d2jb7f.1 Direct URL to data: https://data.mendeley.com/datasets/rgn2d2jb7f/1 Version: 1 License: CC BY 4.0 Instructions for accessing these data: The dataset is publicly available through the URL above.
Related research article	None.

## 1 Value of the Data

• The collection provides 9106 standardized 512 × 512 RGB tuber images distributed across four potato varieties and 18 combined variety-condition classes, with class counts reported explicitly.
• The same acquisition protocol includes five viewing orientations and two physical backgrounds, enabling reuse in studies of viewpoint sensitivity, background dependence, tuber phenotyping, and condition recognition.
• The class folders include healthy, mild, moderate, severe, and, where available, fully rotten visual categories, supporting within-variety ordinal condition studies as well as combined variety-condition classification.
• The data preserve the observed unequal class frequencies rather than artificially balancing the raw image collection, allowing researchers to study methods under class-imbalanced conditions.
• The standardized JPG release and documented acquisition protocol can be reused for computer-vision benchmarking, feature extraction, transfer learning, augmentation studies, and development of agricultural decision-support methods.

## 2 Background

Potato tuber diseases can reduce postharvest quality and storage value. Dry rot is commonly associated with Fusarium species and can produce sunken or wrinkled lesions, dry or leathery tissue, and progressive shriveling [1,2]. Potato common scab is associated with pathogenic Streptomyces species and can produce superficial, raised, corky, or pitted lesions on tuber surfaces [3]. These visible surface changes motivated the use of RGB photography for documenting tuber condition. A recent Data in Brief potato resource focused on leaf imagery for early- and late-blight detection [4]. The present collection was compiled to document tuber-level appearance using four varieties available in Bangladesh. BARI Alu-25 (Asterix) and BARI Alu-7 (Diamant) are documented among Bangladeshi potato varieties [5], while a recent survey of potato varieties cultivated in Bangladesh also reports Rumana and Lal Pakhri as local varieties [6]. The acquisition design records each tuber from multiple orientations and on two backgrounds. The dataset was assembled to provide transparent class labels, class counts, collection conditions, and reusable raw images for image-based agricultural research.

## 3 Data Description

The public dataset contains 9106 processed RGB JPG images standardized to 512 × 512 pixels and arranged into 18 combined variety-condition folders. The class counts are listed in Table 1. Rumana contributes 2526 images; BARI Alu-25 (Asterix), 2379; Lal Pakri, 2254; and BARI Alu-7 (Diamant), 1947. The class distribution is preserved from the retained source collection. Because the source photographs were not square, some released images contain uniform padding bands along the horizontal or vertical edges; these bands were introduced during aspect-ratio-preserving standardization and are not part of the original scene. Representative images from the 18 classes are provided in Fig. 2, while Fig. 1 summarizes the folder hierarchy and class counts.

**Table 1:** Class-wise composition of the potato tuber image dataset.

Variety	Condition / severity class	Image count
Rumana	Healthy	580
Rumana	Scab-Mild	205
Rumana	Scab-Moderate	742
Rumana	Scab-Severe	999
BARI Alu-25 (Asterix)	Fully Rotten	102
BARI Alu-25 (Asterix)	Healthy	510
BARI Alu-25 (Asterix)	DryRot-Mild	142
BARI Alu-25 (Asterix)	DryRot-Moderate	544
BARI Alu-25 (Asterix)	DryRot-Severe	1081
Lal Pakri	Healthy	647
Lal Pakri	DryRot-Mild	61
Lal Pakri	DryRot-Moderate	254
Lal Pakri	DryRot-Severe	1292
BARI Alu-7 (Diamant)	Fully Rotten	141
BARI Alu-7 (Diamant)	Healthy	514
BARI Alu-7 (Diamant)	DryRot-Mild	77
BARI Alu-7 (Diamant)	DryRot-Moderate	330
BARI Alu-7 (Diamant)	DryRot-Severe	885
**Total**		**9106**

**Fig. 1:**
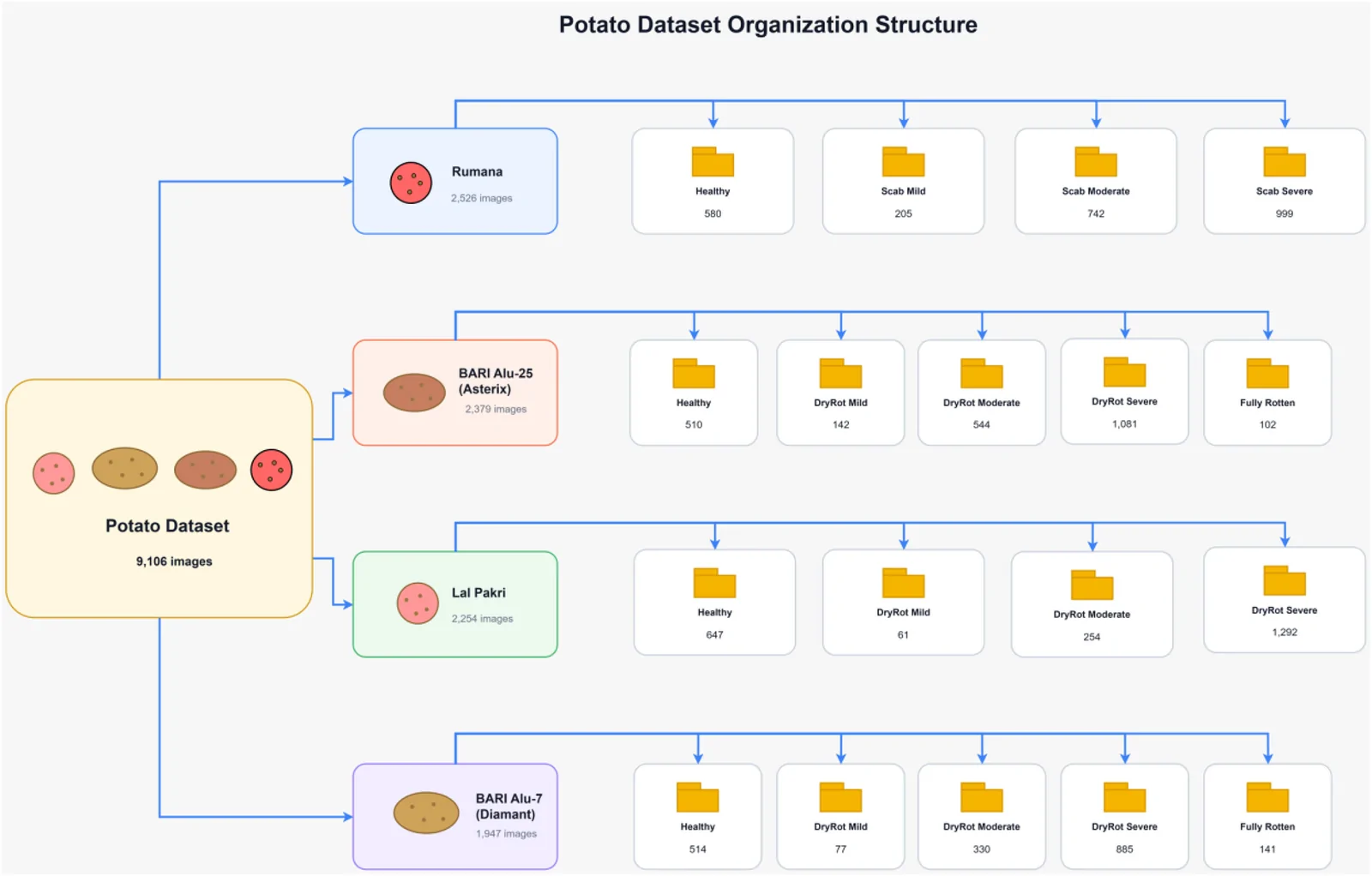
Potato dataset organization structure showing the four varieties, their combined condition/severity classes, and the retained image counts in each class.

The 18 image folders used for public release are:

Rumana__Healthy/

Rumana__Scab_Mild/

Rumana__Scab_Moderate/

Rumana__Scab_Severe/

BARI_Alu_25_Asterix__Healthy/

BARI_Alu_25_Asterix__DryRot_Mild/

BARI_Alu_25_Asterix__DryRot_Moderate/

BARI_Alu_25_Asterix__DryRot_Severe/

BARI_Alu_25_Asterix__Fully_Rotten/

Lal_Pakri__Healthy/

Lal_Pakri__DryRot_Mild/

Lal_Pakri__DryRot_Moderate/

Lal_Pakri__DryRot_Severe/

BARI_Alu_7_Diamant__Healthy/

BARI_Alu_7_Diamant__DryRot_Mild/

BARI_Alu_7_Diamant__DryRot_Moderate/

BARI_Alu_7_Diamant__DryRot_Severe/

BARI_Alu_7_Diamant__Fully_Rotten/

The published Mendeley Data repository package [7] contains the main image archive potato_disease_dataset.zip, comprising the standardized 512 × 512 RGB image release, together with README.md describing the collection and label semantics, potato_dataset_class_counts.csv containing the 18 folder counts, and resize_manifest.csv summarizing the image-standardization settings. The dataset is publicly available as Version 1 under the CC BY 4.0 license at https://data.mendeley.com/datasets/rgn2d2jb7f/1 (doi:10.17632/rgn2d2jb7f.1).

## 4 Experimental Design, Materials and Methods

### 4.1 Sample source and acquisition periods

Potato tubers were obtained from New Kafela Cold Storage, Shibganj, Bogura, Bangladesh. Image collection was conducted during two separate approximately one-week campaigns beginning on 8 November 2025 and 22 July 2026. Four varieties were included: Rumana, BARI Alu-25 (Asterix), Lal Pakri, and BARI Alu-7 (Diamant). A recent Bangladesh survey reports Rumana and Lal Pakhri among local potato varieties [6]; the spelling `Lal Pakri' is retained in this article because it is the class label used throughout the dataset. BARI Alu-25 and BARI Alu-7 are also known as Asterix and Diamant, respectively [5,6].

### 4.2 Manual labeling and visual condition categories

All images were manually reviewed and labeled by the first author, Samiul Islam Saif, using visual phenotypic assessment of the tuber surface. Variety identity, visible condition, and ordinal severity category were assigned during dataset curation. Rumana images were labeled Healthy, Scab-Mild, Scab-Moderate, or Scab-Severe. Asterix, Lal Pakri, and Diamant images were labeled Healthy, DryRot-Mild, DryRot-Moderate, or DryRot-Severe; Fully-Rotten was additionally retained for Asterix and Diamant. No independent plant-pathology reviewer, microbiological culture, molecular assay, or other laboratory etiological test was used. The annotations therefore represent visually assessed phenotypic condition/severity labels rather than laboratory-confirmed diagnoses. Representative examples from all 18 combined variety-condition/severity classes are shown in Fig. 2; the figure is intended to illustrate the visual appearance of the assigned categories rather than to provide diagnostic confirmation. The operational interpretations used for the visual condition/severity labels are summarized in Table 2.

**Fig. 2:**
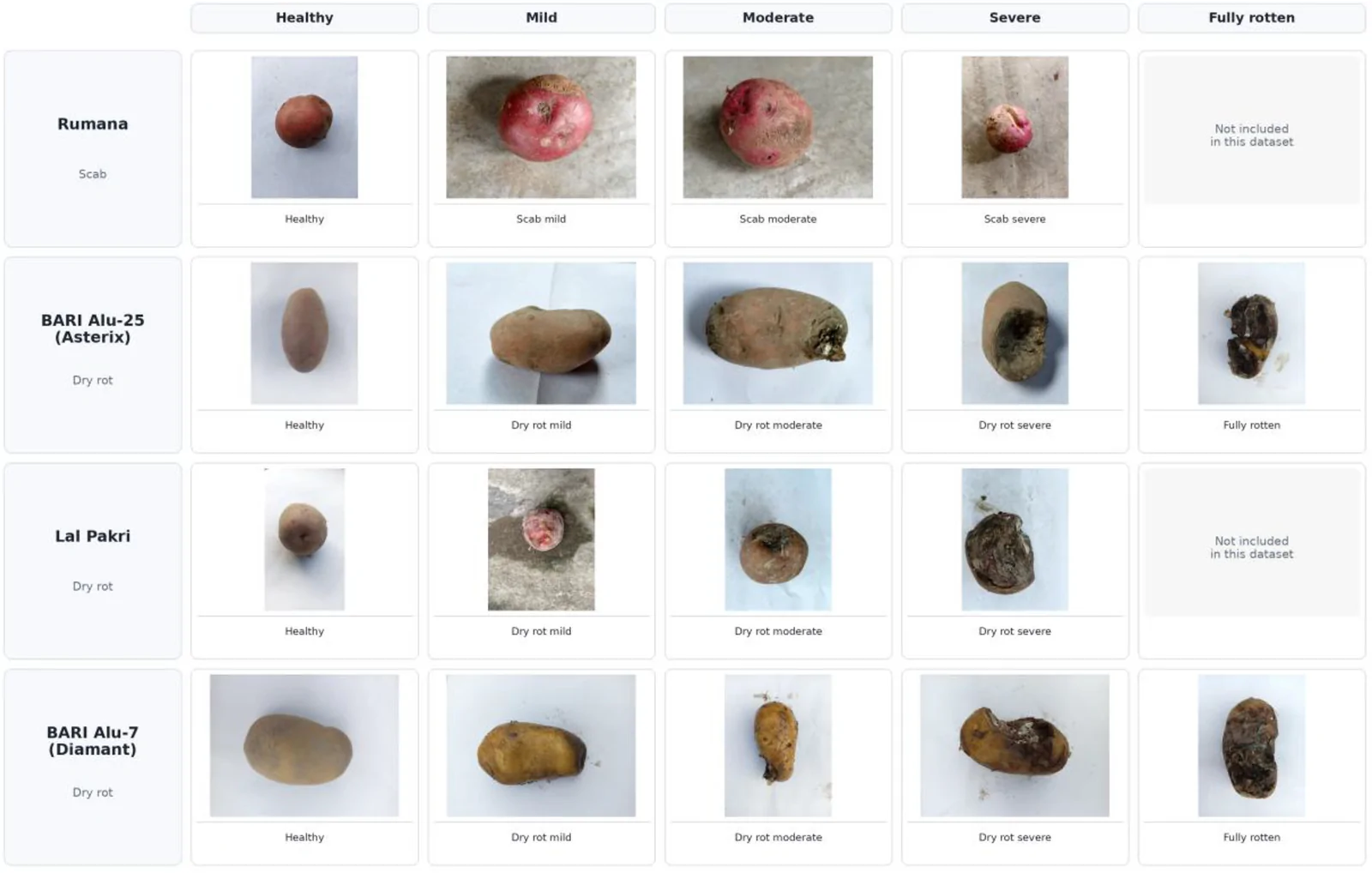
Representative source photographs selected from the 18 combined variety-condition/severity classes. Rumana is represented by Healthy and Scab-Mild, Scab-Moderate, and Scab-Severe categories; BARI Alu-25 (Asterix), Lal Pakri, and BARI Alu-7 (Diamant) are represented by Healthy and dry-rot severity categories, with Fully Rotten additionally present for Asterix and Diamant. The examples illustrate visually assigned phenotypic labels and do not constitute laboratory-confirmed pathogen diagnoses.

**Table 2:** Operational interpretation of the manually assigned visual condition/severity labels.

Visual label	Operational interpretation
Healthy	No obvious visible symptoms corresponding to the condition category used for that variety.
Mild	Limited, localized visible symptoms affecting a relatively small portion of the tuber surface.
Moderate	More developed and spatially broader visible symptoms than Mild, while the tuber remains structurally intact.
Severe	Extensive and visually pronounced symptoms affecting a substantial portion of the tuber, while the overall tuber structure remains recognizable.
Fully Rotten	Advanced visually extensive decay with widespread darkened or necrotic tissue, pronounced tissue breakdown, collapse/shriveling or partial disintegration, and substantial loss of normal tuber structure. Present only for Asterix and Diamant.

### 4.3 Image acquisition protocol

Images were captured with two smartphones, an iQOO Neo 11 and a realme Narzo 50. The source files were stored in JPG format at approximately 4096 × 3072 pixels. Image acquisition was performed under natural daylight with the phone positioned approximately 15–20 cm from the tuber. Two backgrounds were used: white paper and a concrete floor.

For each physical tuber, five orientations were photographed: top, bottom, middle, left, and right. The same tuber was photographed on both backgrounds, providing up to ten photographs per tuber when all views were retained. The final released collection contains 9106 retained images, so not every physical tuber necessarily contributes exactly ten retained files. The overall dataset collection and curation workflow is summarized in Fig. 3.

**Fig. 3:**
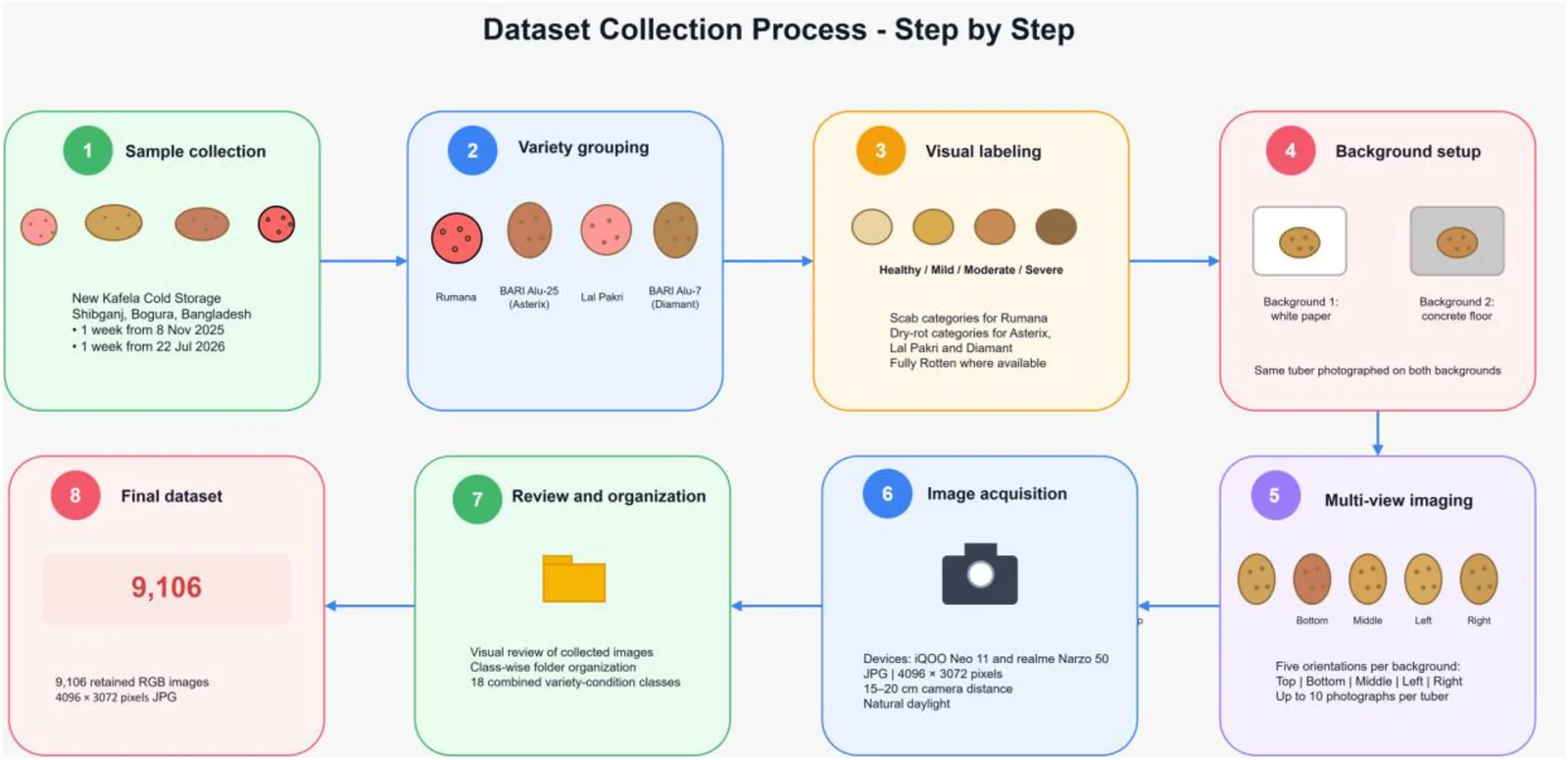
Dataset collection and curation workflow before repository standardization: sample collection, variety grouping, manual visual labeling, dual-background setup, five-view imaging, source-image acquisition, review, and final class-wise organization. The retained source JPG images were subsequently standardized to 512 × 512 pixels for public release as shown in Fig. 4.

### 4.4 Dataset organization and count verification

After acquisition and manual labeling, the retained source JPG files were organized into 18 folders formed by combining variety name with the visual condition/severity label. Class-wise image counts were verified against Table 1. The resulting totals are 2526 images for Rumana, 2379 for BARI Alu-25 (Asterix), 2254 for Lal Pakri, and 1947 for BARI Alu-7 (Diamant), giving 9106 images overall.

### 4.5 Repository-release image standardization

For the public repository release, EXIF orientation metadata were applied and each retained photograph was converted to RGB. Images were then resized with Lanczos resampling while preserving the original aspect ratio and centered on an exact 512 × 512-pixel canvas. The padding color was estimated from four near-corner pixels after a 64 × 64 reduction of the image. The standardized files were saved as high-quality JPEG images (quality 95, optimization enabled, subsampling 0). This procedure avoids geometrically stretching the tuber. As a consequence of preserving the source aspect ratio, uniform padding bands can appear along the left/right or top/bottom edges of some released images; these bands are intentional processing artifacts and should not be interpreted as original scene content. The completed release was verified to contain 9106 images in 18 class folders, with every released image measuring exactly 512 × 512 pixels. The source high-resolution captures are retained separately by the authors; the public repository release described here is the standardized 512 × 512 version. Fig. 4 summarizes this preprocessing and standardization workflow.

**Fig. 4:**
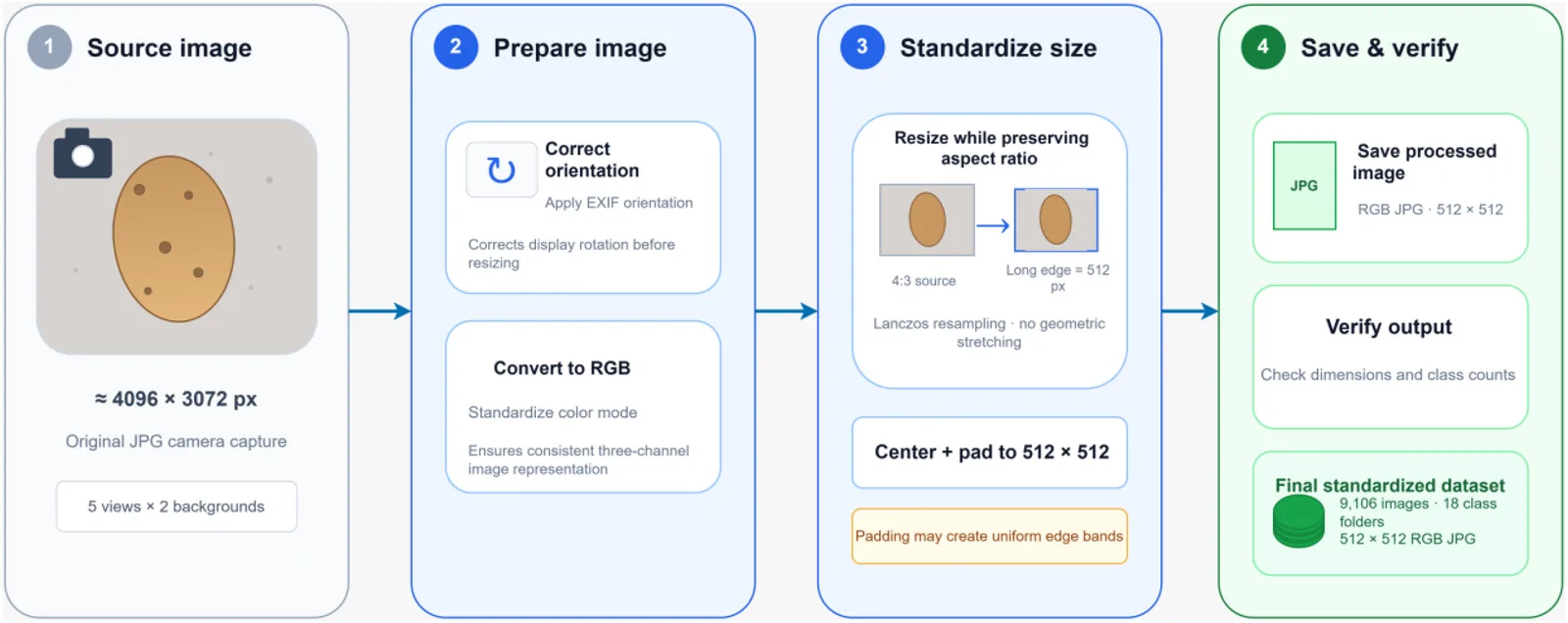
Preprocessing and standardization workflow for the 512 × 512 image dataset. Original approximately 4096 × 3072-pixel JPG images were orientation-corrected, converted to RGB, resized using Lanczos resampling while preserving aspect ratio, centered and padded to 512 × 512 pixels, saved as RGB JPG files, and verified for output dimensions and class counts. Uniform edge bands may occur as a consequence of the padding step.

## Limitations

The collection was obtained from one cold-storage facility in Shibganj, Bogura, which limits geographic and supply-chain diversity. Images were captured under natural daylight with two smartphones, so illumination, exposure, white balance, and device-specific image processing were not standardized as in a laboratory imaging system. The public release is standardized to 512 × 512 pixels and therefore does not preserve the original capture dimensions; aspect-ratio preservation also produces uniform padding bands in some images. Class frequencies are unequal, ranging from 61 Lal Pakri DryRot-Mild images to 1292 Lal Pakri DryRot-Severe images. Disease type is also partly confounded with variety: scab categories occur in Rumana, whereas dry-rot categories occur in Asterix, Lal Pakri, and Diamant. Labels were assigned manually by the first author from visible phenotypic symptoms and were not independently reviewed by a plant-pathology specialist or confirmed through laboratory testing. Fully-Rotten images occur only for Asterix and Diamant. Multiple views and backgrounds can represent the same physical tuber, but stable tuber identifiers are not currently included in the class-folder structure.

## Ethics Statement

The authors have read and follow the ethical requirements for publication in Data in Brief. The current work does not involve human subjects, animal experiments, or data collected from social media platforms.

## CRediT Author Statement

**Samiul Islam Saif:** Conceptualization; Data curation; Investigation; Methodology; Software; Visualization; Writing - original draft. **Mst. Fahmida Tasnim Saba:** Investigation; Validation; Writing - review & editing. **M. Shamim Kaiser:** Supervision; Methodology; Validation; Writing - review & editing.

## Funding

No funding was received for this research.
